# Adherence to antiepileptic drugs among children attending a tertiary health unit in a low resource setting

**DOI:** 10.11604/pamj.2014.17.44.3399

**Published:** 2014-01-22

**Authors:** Rose Nazziwa, Angelina Kakooza Mwesige, Celestino Obua, John M Ssenkusu, Edison Mworozi

**Affiliations:** 1 Department of Pediatrics and Child Health, College of Health Sciences, Makerere University; 2 Department of Pharmacology, College of Health Sciences, Makerere University; 3 Division of Biostatistics, School of Public Health, University of Minnesota, Minneapolis, Minnesota, USA

**Keywords:** Adherence, antiepileptic drugs, children, Epilepsy, Low resource setting

## Abstract

**Introduction:**

Epilepsy is one of the neglected and highly stigmatised diseases, yet it is very common affecting about 70 million people worldwide. In Uganda, the estimated prevalence of epilepsy is 13% with about 156 new cases per 100,000 people per year. Adherence to antiepileptic drugs is crucial in achieving seizure control yet in Uganda; there is lack of information on adherence to antiepileptic drugs and the factors that affect this among children. This study was therefore designed to determine the level of adherence to antiepileptic drugs and the factors that are associated with non adherence.

**Methods:**

In a cross sectional study, 122 children who met the inclusion criteria were enrolled and interviewed using a pretested questionnaire. Assessment of adherence to antiepileptic drugs was done by self report and assay of serum drug levels of the antiepileptic drugs. Focus group discussions were held to further evaluate the factors that affect adherence.

**Results:**

Age range was 6 months - 16 years, male to female ratio 1.3:1 and majority had generalised seizures 76 (62.3%). Adherence to antiepileptic drugs by self report was 79.5% and 22.1% by drug levels. Majority of the children in both adherent and non adherent groups by self report had inadequate drug doses (95/122). Children were found to be more non-adherent if the caregiver had an occupation (p-value 0.030, 95%CI 1.18-28.78)

**Conclusion:**

Majority of children had good adherence levels when estimated by self report. The caregiver having an occupation was found to increase the likelihood of non adherence in a child.

## Introduction

Epilepsy is a disorder of the brain characterised by an enduring predisposition to generate epileptic seizures and by the neurobiological, cognitive, psychological, and social consequences of this condition [1]. It is one of the commonest chronic neurological disorders affecting all people worldwide with no geographical or social boundaries [2]. Prevalence rates of epilepsy are higher in developing countries and range from 2.2 to 58 per 1000 in Africa [3]. The burden of epilepsy in Uganda is not well documented, though the estimated prevalence is 13% and the number of new cases per year is about 156 patients per 100,000 people [2, 4].

The goal of treatment is to maintain a normal lifestyle, free of seizures and with minimal side-effects while on medication [5]. Previous studies have found that 60- 80% of patients with epilepsy are well controlled with anti epileptic drugs yet for this to be achieved, adherence to medication should be observed [5]. Suboptimal adherence levels have been reported with non-adherent patients more likely to have seizures which are associated with increase in number of admissions and healthcare costs [6].

Adherence refers to how patient treatment related behaviors correspond to health professionals’ advice. It portrays greater patient involvement in treatment as well as a mutual arrangement of cooperation and agreement between the health provider and the patient [6]. Rates of adherence to antiepileptic drugs are variable in different studies ranging between 20-80%. In children however, these rates are even lower estimated between 25 - 75% [7, 8]. The adherence rate varies depending on population being studied and method being used, with self report being reported to overestimate adherence. Self report has been the main method of assessing adherence in Low Resource Settings since its affordable and adaptable to study population and unfortunately drug levels have rarely been utilized probably due to costs involved.

Non-adherent patients are more likely to frequently experience seizures which increases the overall cost of health care imposing a financial burden on the caregivers who meet the treatment costs [6, 9– 10]. These patients are frequently hospitalized with prolonged lengths of stay and more emergency department visits [6, 11, 12]. They are also likely to miss school or work because of the seizure effects or out of fear of seizure occurrence [13].

Adherence to antiepileptic drugs may be improved by using a number of interventions such as patient counseling, use of a special medication container, self-recording of medication intake and seizures, and mailed reminders to collect prescription refills and attend clinic appointments [14].

Previous studies have documented a number of factors affecting adherence to antiepileptic drugs such as duration of taking drugs, number of tablets, use of alternative treatment, family support and financial constraints [9, 10, 16]. The type of seizures and their frequency may also motivate patients to adhere to their medication [17].

Adherence to antiepileptic drugs in resource limited settings is not well understood. These settings are prone to frequent drug shortages and with the poverty level in these settings, patients and/or caregivers may not always afford purchasing antiepileptic drugs as prescribed. Our study assesses the level of adherence to antiepileptic drugs in children attending an outpatient clinic and describes the factors that affect adherence in a resource limited setting.

## Methods

### Study design

**Figure 1:**
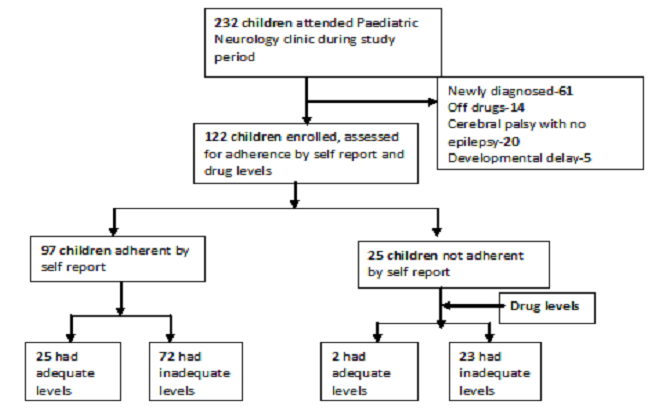
Study profile

A cross sectional study was conducted at Mulago hospital Peadiatric Neurology clinic between December 2010 and March 2011. Mulago hospital is the national referral hospital of Uganda, located in Kampala the capital city. It receives referrals from health facilities throughout the country with a few self referrals. The pediatric neurology clinic is an outpatient clinic that treats patients < 19 years old with different neurological conditions such as epilepsy, cerebral palsy and developmental delays (Figure 1).

### Study Population and sampling

Children with epilepsy, aged between 2 months to 18 years who attended the pediatric neurology clinic during the study period were enrolled into the study.

A minimum sample size of 121 patients was calculated using the Daniel, 1999 formula for finite populations, assuming an estimated adherence to antiepileptic drugs at 15.5% (Odaga et al 2008), precision of 5% (0.05) and an available population of 300 patients with 95% confidence limits.

### Study procedure

Children were identified using daily clinic records, assessed for eligibility and enrolled into the study. They were eligible for the study if they were between 2 months and 18 years old, attended the clinic during the study period, had confirmed epilepsy defined as 2 or more unprovoked seizures, and had been on antiepileptic drugs for at least 4 weeks prior to the clinic visit. Children with epilepsy who were on newer antiepileptic drugs such as Lamotrigine were excluded from this study because the therapeutic drug levels could not be assessed in the available laboratory.

A pretested structured questionnaire was then administered by a trained nurse or a doctor. This questionnaire assessed adherence by self report over the past 3, 7 and 30 days and solicited for factors that affected patient adherence. For children aged 8 years and above self report was solicited from them as well as their caregivers, in case of disagreement, child's report was considered Three focus group discussions were also conducted by a social scientist, two focus groups were for the caregivers (both men and women) and the other was for older children (age ≥ 8 years). Participants in the focus groups were selected from caregivers or children who attended the clinic, fulfilled the inclusion criteria but had not been recruited before in the study.

Under aseptic techniques, 2 mls of blood were drawn from the children on the same day the interview was conducted, from a peripheral vein and stored in a labelled plain vacutainer without any anticoagulant.

The blood samples were then transported to the Lancet laboratories within 8 hours of collection. They were centrifuged at 3000rpm for 10min, serum pippeted off, and analysed by Fluorescence polarisation immunoassay (FPIA) method using cobas^®^ 4000 analyzer series from Roche Diagnostics to quantify the drug levels.

### Data handling and analysis

Data was checked for completeness, coded and entered into a computer using Epidata version 3.1 packages and analyzed using STATA version 10.0 (STATA Corporation, College Station, Texas, USA). Adherence to antiepileptic drugs was summarized as the proportion of patients who took more than 85% of their prescribed doses by self report and the proportion of children who had adequate serum drug levels. The cutoff of 85% for adherence by self report was based on a study by Lisk et al [18] who noted that at this level of self reported adherence most patients had adequate drug levels and this correlated with seizure control. The Chi-square test or Fisher's exact test were used to determine factors associated with adherence and we report odds ratios and their 95% confidence intervals. Multivariate analysis was conducted using logistic regression and a p-value ≤ 0.05 was taken to be statistically significant. Data from the focus group discussions was analyzed using the content thematic approach.

### Ethical issues

Before enrollment, written informed consent was obtained from all the caregivers and assent from children aged 8years and above. Ethical approval for the study was obtained from the department of Paediatrics and Child health of Makerere University, Makerere University College of Health Sciences Research and Ethics Committee and the management of the Paediatric Neurology Clinic. The consent and assent forms were availed in both English and Luganda which are the commonest languages in the area

## Results

The study enrolled 122 children and there were equal numbers below and above 5 years with a mean age of 6 years. Most children 62.3% (76/122) had generalized seizures. Four children (3.3%) were orphaned but with at least one parent. More than half (65.6%) of the children not attending school were below 4 years, 13 (20.3%) had mental retardation and 7 (10.9%) were sickly with very frequent seizures. Among those attending school 41/58 (71%) were in primary school level, 15 (26%) in Nursery and only 2 (3%) in secondary school level.

Ninety eight children (80.3%) were on monotherapy of which 73.5% of the children were taking carbamazepine and the rest taking valproate. Of the 24 children on combination therapy only one was on three drugs (Carbamazepine, Valproate and Phenobarbitone), the rest were on two drugs either Carbamazepine and Valproate (12/23) or Carbamazepine and Phenobarbitone (11/23).

Mothers were the majority care givers (64.8%) and most of the care givers were between 25 - 45 years of age (76.2%). Most caregivers were in unskilled employment such as market vendors, shopkeepers, and peasant farmers (53.7%). Only 2 care givers were members of an epilepsy support group from which they received counseling and guidance in caring for their children; the rest had not heard of such groups at the time of the study.

One third of the caregivers had received counseling from the Pediatric Neurology clinic before starting antiepileptic drugs. Doctors were the major providers of counseling and they addressed the nature of child's illness and the importance of taking drugs.

### Adherence to Antiepileptic drugs

The overall adherence by self report was 79.5% (95%CI 72.2% - 86.8%). The proportion of study participants adherent to medication in the past 3, 7 and 30 days was 81.2%, 85.3% and 93.4% respectively. There was variation in mean adherence for all study participants for the different periods assessed; at 3 days it was 88.3%, at 7 days 90.6% and 96.4% at 30 days.

The overall proportion of adherent participants as characterized by drug levels was 22.1% (95%CI 14.8%-29.5%). Only 19 children (15.6%) had adequate drug levels for all drugs taken, 8 (6.6%) had adequate drug levels for at least one of the drugs taken. Seven children (5.7%) had drug levels above the recommended therapeutic drug levels but none of these reported any known side effect to the drugs. Most children 72/97 (74.2%) had inadequate drug levels despite having reported adherence by self report equal to or greater than 85%.

Majority of the children in both adherent and non adherent groups as characterized by self report had inadequate drug doses (95/122).

**Figure 2:**
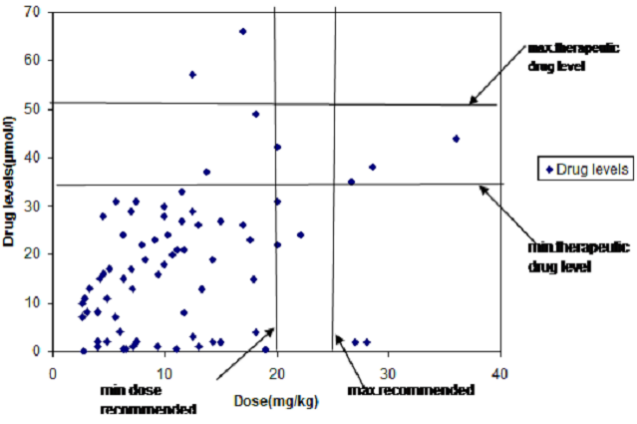
Scatter plot of Carbamazepine drug doses vs. drug levels

There was variability in drug doses and drug levels noted in the study participants, as shown in the scatter plot in Figure 2. Carbamazepine was used in the scatter plot as a representative of the other drugs since it was the commonest drug taken on monotherapy by over 70% (72/122) of the children.

From scatter plot, most of the children 82% (59/72) had below the recommended drug doses hence drug levels below the therapeutic range. One child (1.4%) on Carbamazepine had both the recommended drug doses and drug levels in the therapeutic range while 2 children (2.8%) had drug doses below the recommended but drug levels within the therapeutic range. There were three children (4.1%) who despite having adequate drug doses had drug levels below the recommended therapeutic range. Two children (2.8%) had drug doses below the recommended but drug levels above the recommended therapeutic range.

### Factors affecting adherence to antiepileptic drugs

There were more children who were non adherent and lacked knowledge of convulsions compared to the adherent. The proportion of children who had spent less than 2 years on drugs was higher among the non adherent. These associations were however not significant. No major differences were noted in the child's parent being alive and school attendance between adherent and non adherent children. A higher proportion of children who were non adherent had history of having ever used alternative treatment compared to the adherent ones. There was no difference in the proportions of children who had side effects among the adherent and non adherent ones (Table 1).

**Table 1:** Factors affecting adherence to antiepileptic drugs

Characteristic	Adherent N = 97	Non adherent N = 25	Odds ratio [CI]	p-value
**Age of child:**				
<10 years	72(74.2%)	21(84%)		
≥10 years	25(25.8%)	4(16%)	0.55[0.17-1.75]	0.311
**Type of convulsions**				
Generalized	61(62.9%)	15(60%)		
Not generalized	36(37.1%)	10(40%)	1.13 [0.46-2.78]	0.820^a^
**Duration of AEDs^b^**				
<2years	72(74.2%)	22(88%)		
>2years	25(25.8%)	3(12%)	0.39 [0.11-1.43]	0.155
**Knowledge of convulsions**				
Yes	32(33%)	4(16%)		
No	65(67%)	21(84%)	2.58 [0.82-8.16]	0.106
Number of tablets taken/day*				
<1	51(53.7%)	17(68%)		
≥1	44(46.3%)	8(32%)	0.54 [0.21-1.38]	0.202
**Ever used alternative treatment**				
No	58(59.8%)	10(40%)		
Yes	39(40.2%)	15(60%)	2.23 [0.91-5.47]	0.080

a Fisher's exact test

b AEDs- antiepileptic drugs

* Computed for 120 children, 2 were on syrups. Variables with a p-value < 0.2 were entered into logistic regression model

There were more non adherent children with the primary caregiver as a mother compared to the adherent. The proportion of caregivers less than 35 years was higher for the non adherent compared to the adherent (Table 2). There were no significant differences in caregiver income and education for the adherent and non adherent children.

**Table 2:** Caregiver factors affecting adherence to antiepileptic drugs

Characteristic	Adherent N = 97	Non adherent N = 25	Odds ratio [CI]	p-value
**Caregiver age**				
<35years	57(58.8%)	17(68%)		
>35years	40(41.2%)	8(32%)	0.67[0.26-1.70]	0.401
**Caregiver Relationship**				
Mother	59(60.8%)	20(80%)		
Other	38(39.2%)	5(20%)	0.39 [0.13-1.12]	0.081
**Caregiver education**				
Primary and below	34(35%)	9(36%)		
Above primary	63(64.9%)	16(64%)	0.95 [0.38-2.40]	0.929
**Caregiver occupation**				
None	20(21.6%)	2(12%) 23(88%)		
Employed	77(78.4%)		2.99 [0.65-13.74]	0.160
Caregiver Income*				
< 250,000	69(71.1%)	19(76%)		
>250,000	28(28.9%)	6(24%)	0.78 [0.28-2.15]	0.803

* Computed for only 100 caregivers whose income was known. Variables with a p-value < 0.2 were entered into logistic regression model

The caregiver having an occupation was found to increase the likelihood of non adherence in a child by 5.8 times. Having a primary caregiver who is not the mother reduced the likelihood of non-adherence (Table 3)

**Table 3:** Multivariate analysis of independent predictors of adherence*

Variable	a cOR (95%aCI)	P value	b aOR (95%CI)	P value
**Child having**				
**knowledge of**	2.58 (0.82-8.16	0.106	3.29 (0.73-14.84)	0.122
**convulsions**				
**Having a primary**				
**caregiver not the**	0.39 (0.13-1.12)	0.081	0.25 (0.08-0.76)	0.015
**mother**				
**Caregiver having an occupation**	2.99 (0.65-13.74)	0.160	5.83 (1.18-28.78)	0.030

* Adjusted for age of the child

a cOR- crude odds ratios

b aOR-adjusted odds ratios, CI-confidence intervals, P<0.05 is statistically significant. The above were the variables that remained in the final model.

The commonest reason given for missing drugs was lack of drugs due to their high cost as reported by 36(48.7%) of study participants. This was followed by forgetting, reported by 22(29.7%) of study participants. Only 3 children had missed drugs because they had been advised to stop by either health workers or family members since they no longer had convulsions and had fear of side effects.

Three focus group discussions were held; two groups for caregivers and one for children aged above 8years old without mental retardation. The care givers comprised of 7 participants aged between 25-35 years most of whom had a source of income in form of a business or farming. The children's group comprised of 6 participants aged between 8-12 years who were all attending school.

Factors that were reported to enhance adherence to AEDs include; Availability of drugs, Involvement of family members in care and good health as a motivation for adherence. This was further emphasized by one caregiver and a child;

*“..even when I am travelling to the village, party or any long distance, I travel with the child so that I can give the drugs on time since there is marked improvement in the child's condition from the time of start of drugs”* (Female Caregiver)

*“..I used to get headaches and falls and could not go to school, now I am better therefore I have to continue taking so that I do not miss school again”* (10 years old boy)

On the other hand the reasons given for missing drugs include; Failure to get drugs from the clinic, Inadequate counseling and awareness about duration of taking antiepileptic drugs; beliefs and perceptions regarding antiepileptic drugs and Forgetting to take/administer medication. One child was quoted

*“..I leave home very early for school and I realize that I did not take my drugs when I am already at school so I miss the morning dose”*. (11 years old girl)

All Focus group discussants proposed availing all the drugs at the hospital pharmacy as a way of improving adherence as well as provision of adequate information on epilepsy and the drugs.

## Discussion

### Statement of principal findings

The level of adherence to antiepileptic drugs by self report was 79.5% and 22.1% by drug levels. Most of the children (88/122) had sub-therapeutic drug levels mainly attributed to the inadequate dosing that was found in 78% of these children. Some children had recommended (4.1%) or even above the recommended (2.8%) doses of Carbamazepine but had sub-therapeutic drug levels. Some children despite having inadequate drug doses of Carbamazepine had drug levels within the therapeutic range (2.8%) or even above the therapeutic range (2.8%).

The factor that was found to independently predict adherence was caregiver having an occupation. Having a primary caregiver other than the mother was associated with a reduced likelihood of non-adherence. Duration of epilepsy, number of tablets and use of alternative treatment were not found to be statistically significant in affecting adherence to antiepileptic drugs. The main reasons for missing drugs were lack of drugs due to their high cost and forgetting. Forgetting medication schedules by patients and caregivers was reported by 29.7% during individual interviews and highlighted in the focus group discussions.

### Discussion of important differences in results

Adherence by self report is much higher than what Odaga et al [15] reported using appointment attendance probably due to differences in the method used and age group of study participants. It is however similar to what Modi et al reported using Medication Event Monitoring Systems [19] probably due to similarities in age of study participants despite differences in methods of assessment. It is also comparable to what Asaadi-Pooya et al found using self report [20] due to a similar method of assessment and ages of children as in our study. This high adherence rate by self report may however be spurious given the limitations of self report as a measure of adherence [9]. There was variation in average adherence over 3 days, 7 days and 4 weeks recall which is attributed to recall bias especially being worse for longer durations of recall. This is in agreement with Angelia et al who noted that patients tend to give socially desirable responses thus leading to over estimation of adherence by self report over time [21].

The inadequate dosing noted in our study participants limits the ability to use serum drug levels of antiepileptic drugs as a method of assessing adherence and calls for a review of the children's doses especially as they gain weight over time. Some children had sub-therapeutic drug levels which could be due to actual non adherence to antiepileptic drugs or variation in drug absorption and metabolism. Some children having above or within the recommended range despite under dosing could be attributed to variability in individual pharmacokinetics of the different antiepileptic drugs [22, 23]. Interracial differences in response to drugs have been suggested for several polymorphisms that might be responsible for the differences in serum drug levels and optimal dose requirement for efficacious treatment [24]. This may explain the differences in therapeutic drug levels from our study compared to other studies. [25–27].

It however poses a question regarding the therapeutic drug doses and levels for our population since they may be different from the available ranges done in European populations whose environmental and genetic characteristics are different.

The caregiver having an occupation contributes to non adherence probably because it affects the availability and supervision of medication taking by the child. It was also noted in the focus group discussions where having another family member involved in the care of the child was reported to enhance adherence as they help in drug administration during absence of the primary caregiver. This is similar to what has been found by Helvi who noted that adolescents with parental support were more likely to adhere to their medication [28, 29].

Duration of epilepsy, number of tablets and use of alternative treatment were not found to be statistically significant in affecting adherence to antiepileptic drugs similar to what has been found in other studies on adherence by Asaadi-pooya and Jones, respectively [20, 30] but contrary to Helvi [28]. Use of alternative treatment was found to be common before start of antiepileptic drugs and all caregivers denied using it at the time of interview which might account for the lack of statistical significance in our study. Socioeconomic, racial and family factors have been reported to affect adherence by different studies [19, 26] but these were not significant in our study probably because majority of the caregivers had income within the same category and all were from the same race.

In this study we found that having a primary caregiver other than the mother was associated with a reduced likelihood of non-adherence which is different from other studies. This could be attributed to the small numbers of children who had other caregiver in our study with only a few being non adherent(5/43) However, this finding needs further investigation with sufficient numbers of other primary caregivers other the mother.

The main reasons for missing drugs were similar to what has been reported by Faris et al and Snodgrass et al respectively [16, 27]. The cost of drugs limits caregivers from purchasing them in case they are not availed at the clinic due to other priorities such as rent and food which usually override purchasing of medication thus leading to non adherence. Competing priorities such as food and rent being prioritised over purchase of medication has also been reported by Modi et al as a reason for missing drugs [19].

Forgetting medication could be attributed to improvement in the children's condition leading to reduction in perceived risk of convulsions as has been reported by Faris et al who found that parents who perceived their children as not susceptible to convulsions were less likely to adhere to medication [16]. It could also be due to caregivers having busy work schedules as was noted that 92% of the caregivers had some form of employment which limits their availability to supervise medication intake.

Some children missed drugs because they had been advised to stop medication by either health workers or family members due to perceived fear of side effects following long duration of treatment or marked improvement with no seizures in more than 6 months. These highlight the gaps in epilepsy management in terms of duration of treatment and when to stop drugs for both health workers and the community who seem not to follow clear guidelines before stopping medications.

### Strengths and Weaknesses of the study

The study strength is the evaluation of adherence using drug levels which is a more objective measure for all study participants in addition to the self report. The use of focus group discussions in addition to the individual interviews also led to a better understanding of reasons for non adherence and enabled getting participants’ ideas on how this could be improved.

We are however limited by the use of Self report since it is prone to recall bias especially where caregivers alone report on adherence and are likely to overestimate it. In addition, one assay for drug levels was done which might not reflect the variation in drug levels by the day but since these clients had been on drugs for 4weeks and more, it was expected that they had attained the therapeutic ranges if they were adherent.

### Meaning of the study

Adherence to antiepileptic drugs in children is suboptimal, varying with the method of assessment. The factors that affect adherence can be addressed with simple interventions such as reminders.

### Future research

It should be carried out to establish the baseline effective concentration and doses for children with epilepsy in our setting.

## Conclusion

Adherence to antiepileptic drugs among children with epilepsy attending the paediatric neurology clinic at a National referral hospital as assessed by self report was good, at 80% and by drug levels it was low at 22%. The caregiver having an occupation was found to be associated with poor adherence and having another primary caregiver other than the mother was found to be associated with good adherence. The common reasons reported for missing drugs were Lack of drugs due to their high cost, forgetting and inadequate information concerning drugs. Clinicians should ensure regular weighing and review of drug doses so as to promote adequate dosages for children with epilepsy.
